# Supplementary material for: Broad ultra-potent neutralization of SARS-CoV-2 variants by monoclonal antibodies specific to the tip of RBD
Source: Cell Discov. 2022 Feb 15;8:16. doi: 10.1038/s41421-022-00381-7 (PMC8847360; doi:10.1038/s41421-022-00381-7)
Supplement: Supplementary file 1 — Supplementary Information [file 41421_2022_381_MOESM1_ESM.pdf]

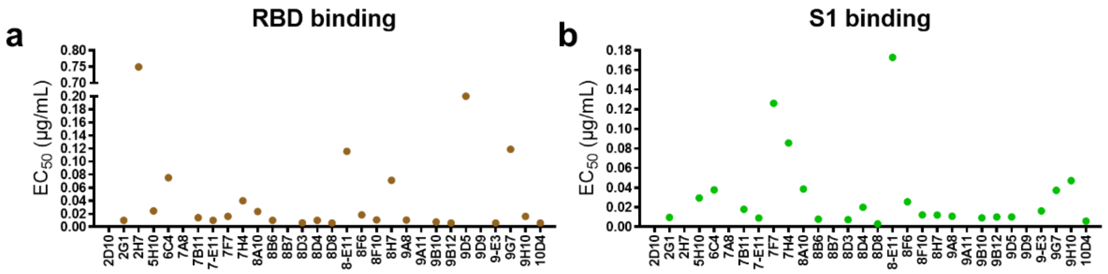

Supplementary Fig. S1 Evaluation of binding and neutralization of selected antibody candidates. a-b, Candidates' EC_{50} in the concentration-dependent RBD (**a**) and S1 (**b**) binding test using ELISA. Antigens were 3-fold serially diluted from 0.300 μg/mL to 0.0012 μg/mL.

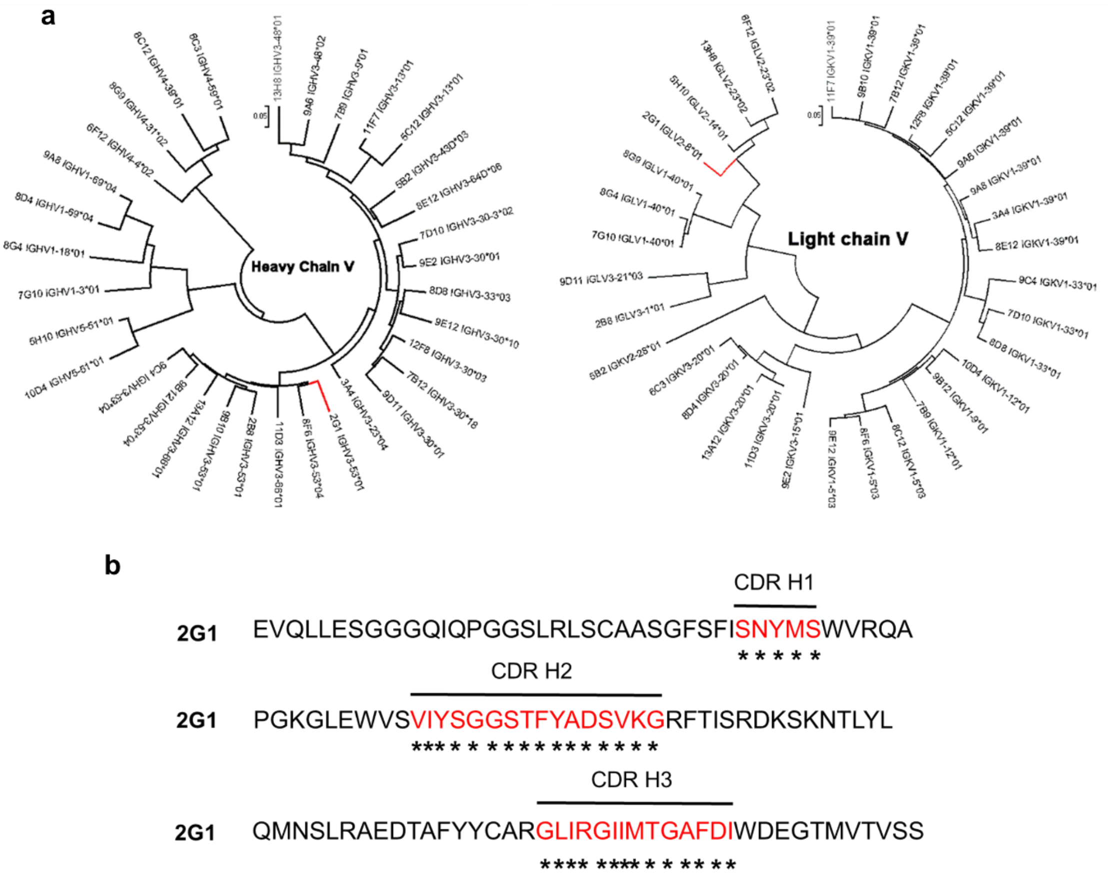

Supplementary Fig. S2 Germline identification of VH and VL. a, Germline gene distribution of the heavy chain and light chain of 33 candidates and their clustering analysis. **b,** The heavy-chain variable domain of 2G1.

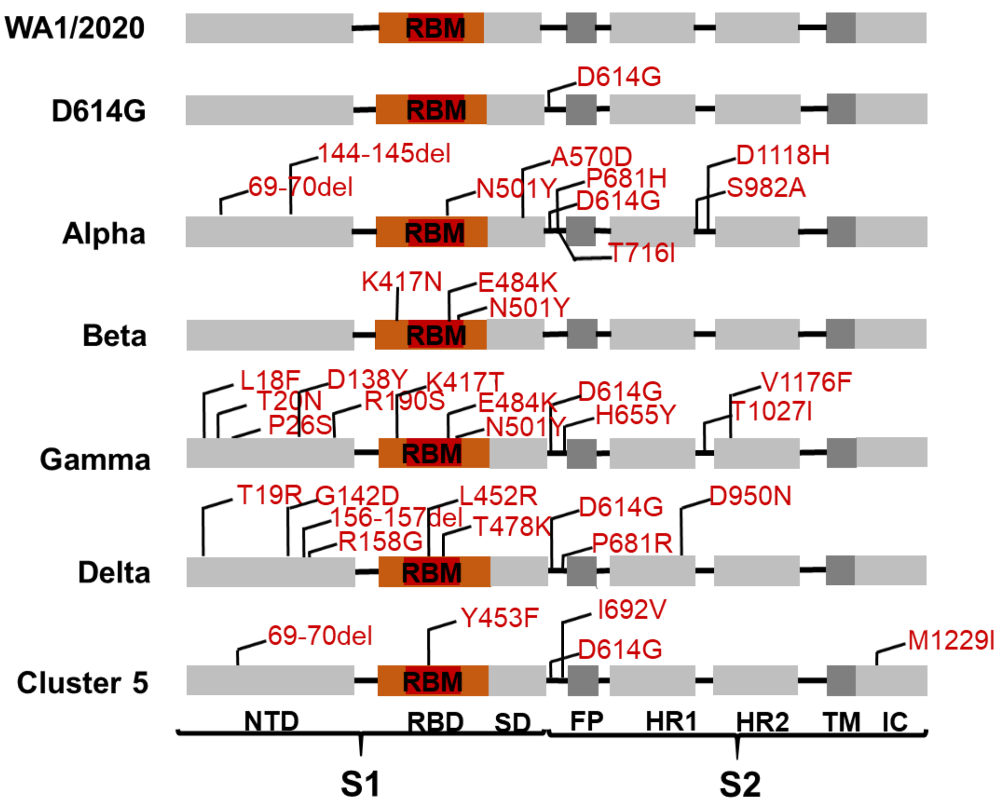

Supplementary Fig. S3 Mutational sites of pseudoviruses used in this report. The spike region of SARS-CoV-2 is displayed in different modules. The mutation sites are annotated in corresponding positions in detail. RBD is highlighted in saffron yellow and RBM is highlighted in red. NTD, N-terminal domain; RBD, receptor binding domain; RBM, receptor binding motif; SD, subdomain; FP, fusion peptide; HR1, heptad repeats 1; HR2, heptad repeats 2; TM, transmembrane region; IC, intracellular region.

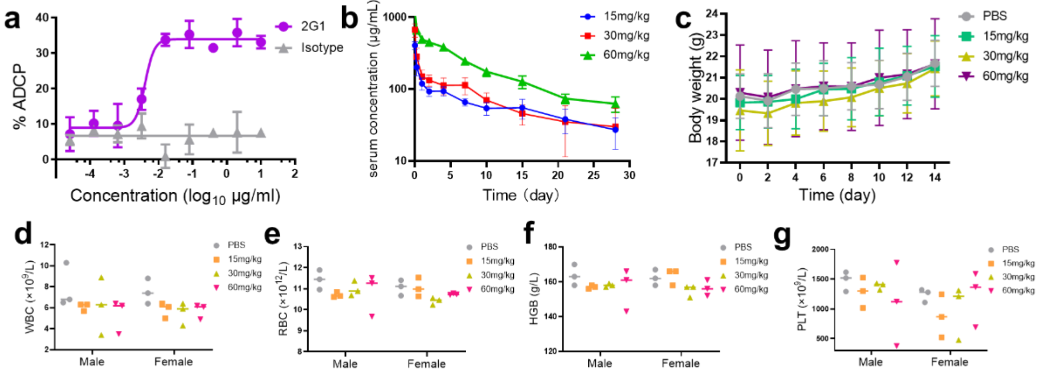

Supplementary Fig. S4 2G1 induces cellular phagocytosis but no evident adverse effects. a, Antibody-dependent cellular phagocytosis (ADCP) induced by 2G1. Jurkat cells with stable S expression were incubated with macrophages in the presence of different concentrations of 2G1. After incubating at 37 ℃ for 30 mins, the proportion of Jurkat cells phagocytosed by macrophages was detected by flow cytometry. **b,** Pharmacokinetic study of 2G1. BALB/c mice were treated with different doses of 2G1, and blood samples were collected at different time points. The serum concentration of 2G1 was measured by ELISA. **c-g,** Adverse effect study of 2G1. Crlj:CD1(ICR) mice were treated with different doses of 2G1. Body weight of mice was tracked (**c**). The blood routine indexes including WBC (**d**), RBC (**e**), HGB (**f**), and PLT (**g**) were measured 14 days later. WBC, white blood cell count; RBC, red blood cell count; HGB, hemoglobin; PLT, platelets. Data are presented as mean ± S.D.

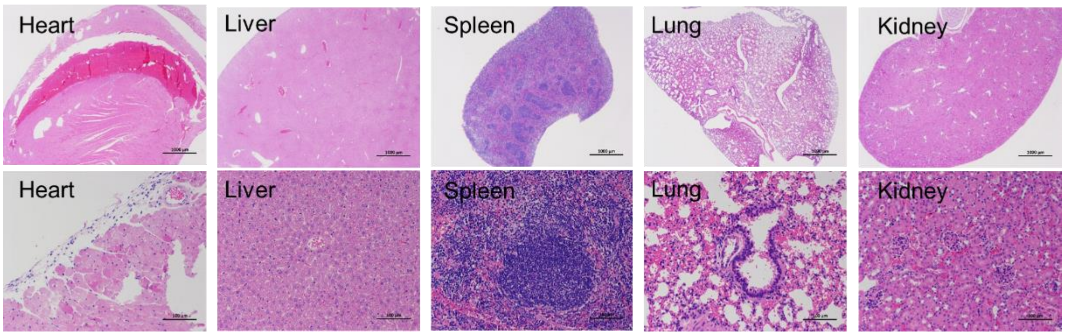

Supplementary Fig. S5 Organ toxicity study. Crlj:CD1(ICR) mice were treated with 15, 30, or 60 mg/kg of 2G1. Inflammatory damage of hearts, livers, spleens, lungs and kidneys were checked by hematoxylin-eosin (HE) staining. No apparent pathological changes were observed. Representative sections from 60 mg/kg group are displayed. Scale bar, upper 1,000 μm, under 100 μm.

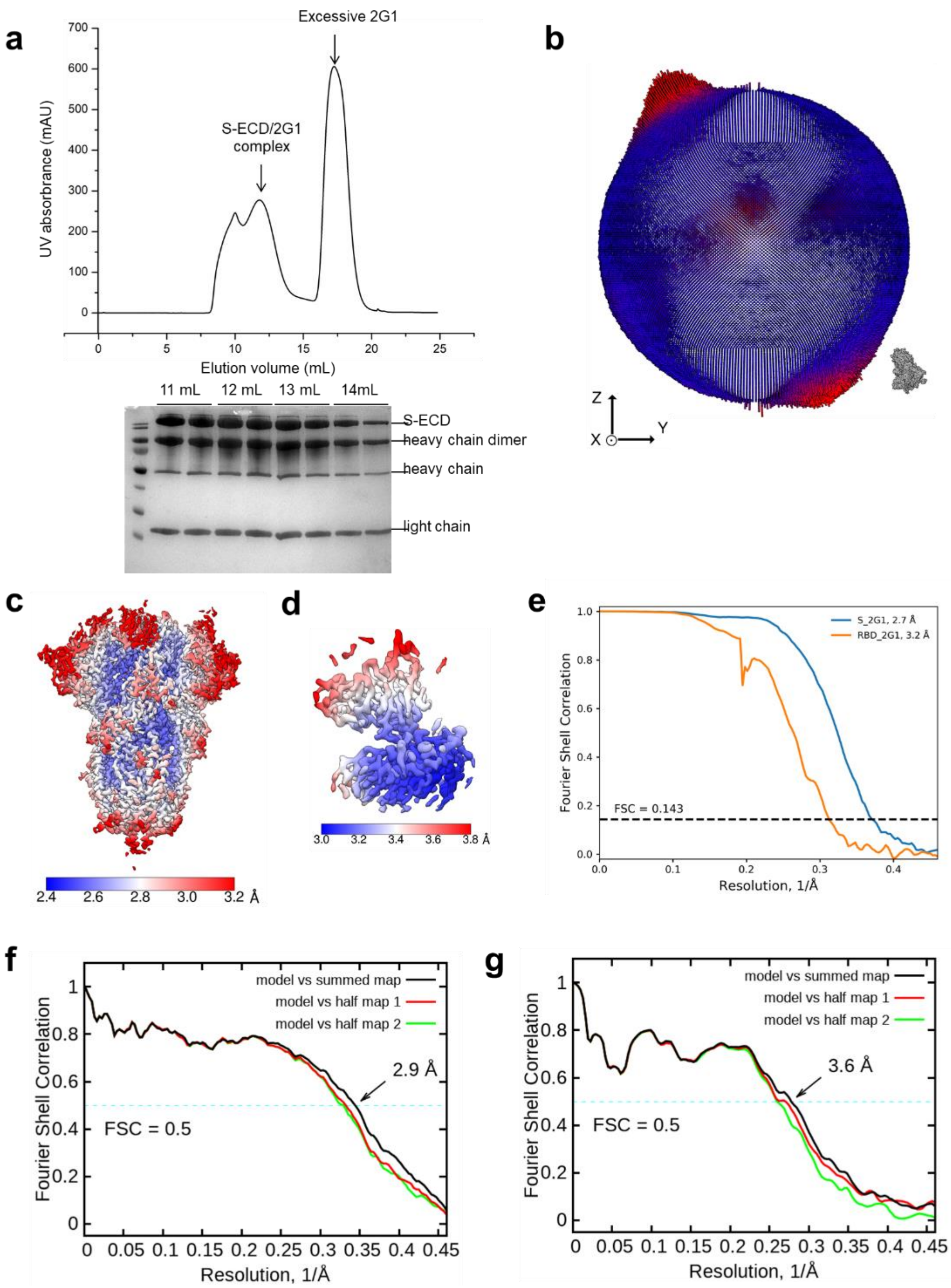

Supplementary Fig. S6 Cryo-EM analysis of SARS-CoV-2 S trimer in complex with 2G1. a, Representative gel filtration chromatography purification profile of the SARS-CoV-2 S extracellular domain in complex with 2G1. **b**, Euler angle distribution in the final 3D reconstruction of S bound with 2G1. **c-d,** Local resolution map for the 3D reconstruction of overall structure and RBD-2G1 sub-complex, respectively. **e,** FSC curve of the overall structure (blue) and RBD-2G1 sub-complex (orange). **f,** FSC curve of the refined model of S bound with 2G1 versus the overall structure that it is refined against (black); of the model refined against the first half map versus the same map (red); and of the model refined against the first half map versus the second half map (green). The small difference between the red and green curves indicates that the refinement of the atomic coordinates did not suffer from overfitting. **g,** FSC curve of the refined model of RBD-2G1 sub-complex, which is same to the **f**.

a

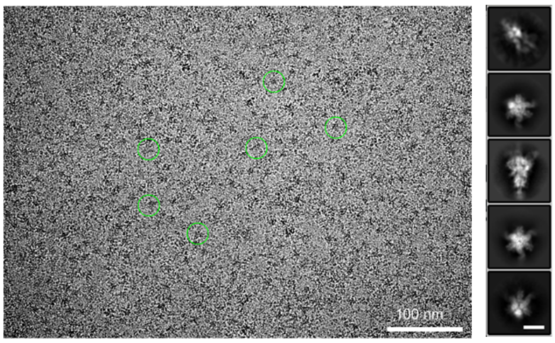

b

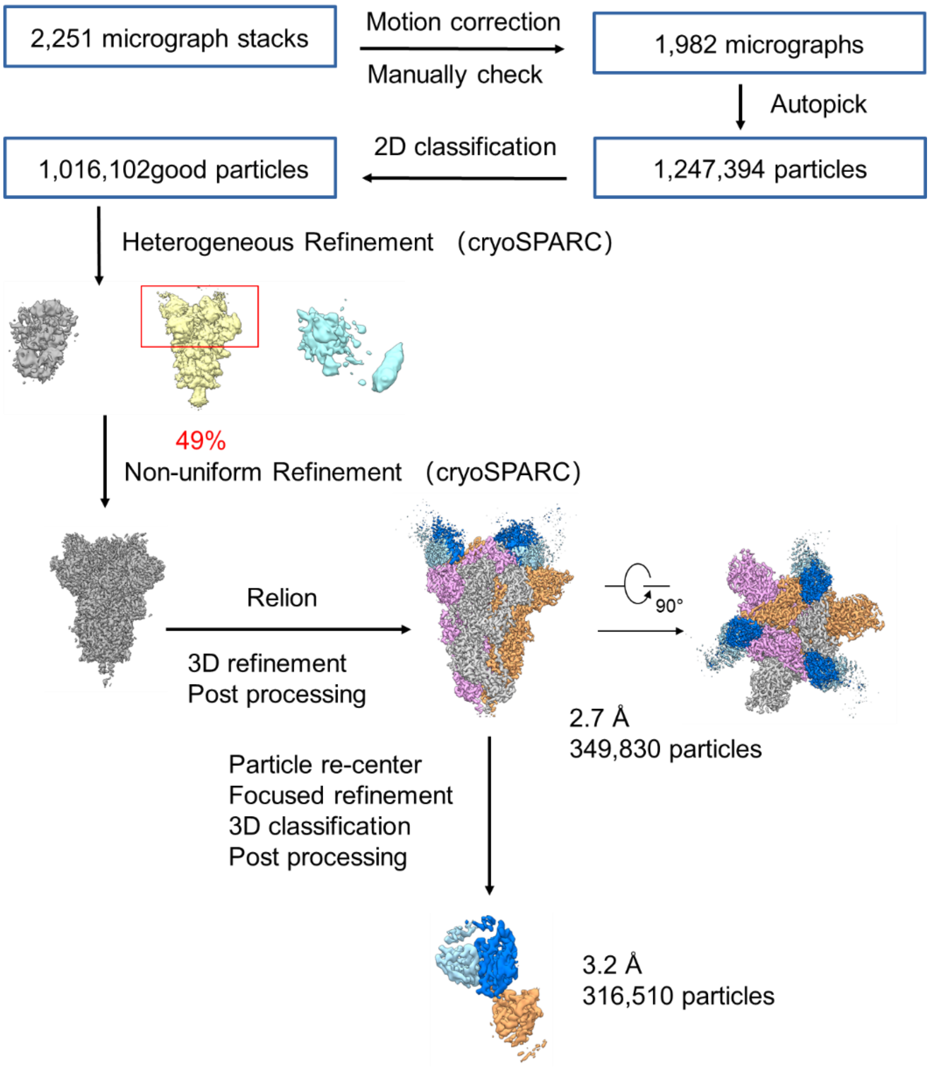

Supplementary Fig. S7 Flowchart for cryo-EM data processing of SARS-CoV-2 S trimer in complex with 2G1. a, Representative cryo-EM micrograph and 2D class averages of cryo-EM particle images of **SARS-CoV-2 S trimer** bound with 2G1. The scale bar in 2D class averages is 10 nm. **b,** Please refer to the 'Data Processing' in Methods section for details.

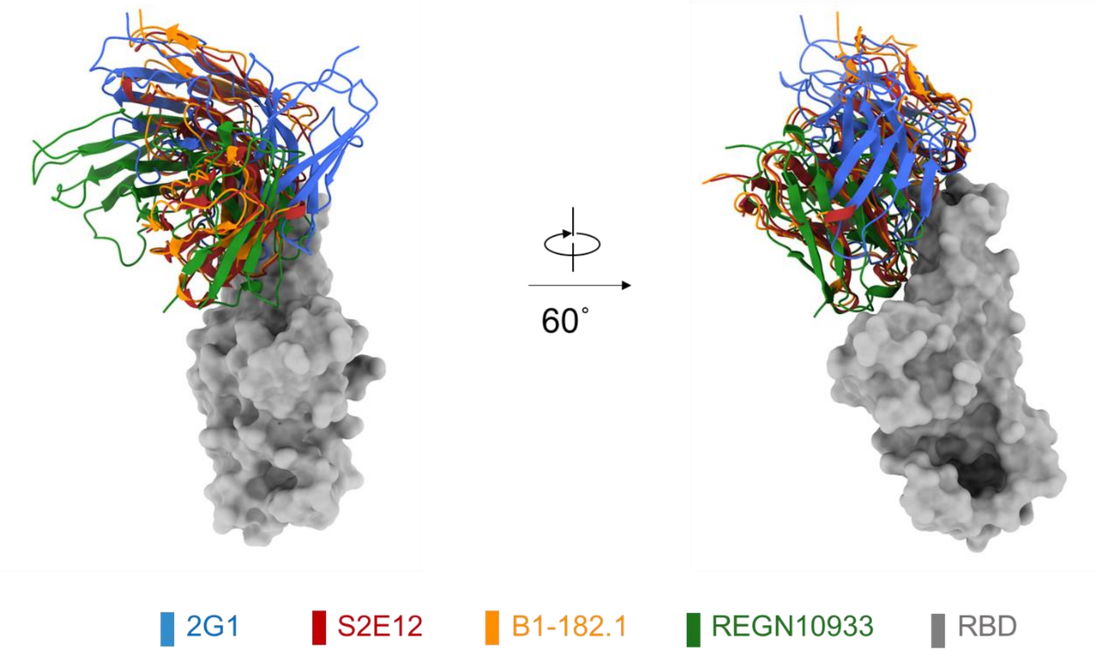

Supplementary Fig. S8 Structural alignment of 2G1, S2E12, B1-182.1 and REGN10933. 2G1, S2E12, B1-182.1 and REGN10933 are colored as black, blue, red, orange and green respectively.

Supplementary Table S1 Data collection, 3D reconstruction and model statistic.

**Data collection**		
EM equipment	Titan Krios (Thermo Fisher Scientific)	
Voltage (kV)	300	
Detector	Gatan K3 Summit	
Energy filter	Gatan GIF Quantum, 20 eV slit	
Pixel size (Å)	1.087	
Electron dose (e-/Å2)	50	
Defocus range (μm)	-1.2 ~ -2.2	
Number of collected micrographs	2,251	
Number of selected micrographs	1,982	
Sample	S protein in complex with 2G1	
**3D Reconstruction**		
	Whole model	Interface between RBD and 2G1
Software	cryoSPARC/ Relion	Relion
Number of used particles	349,830	316,510
Resolution (Å)	2.7	3.2
Symmetry	C1	
Map sharpening B factor ($Å^2$)	-90	
**Refinement**		
Software	Phenix	
Cell dimensions (Å)	313.056	
Model composition		
Protein residues	4,572	
Side chains assigned	4,572	
Sugar	78	
Linoleic acid	3	
R.m.s deviations		
Bonds length (Å)	0.007	
Bonds Angle (˚)	0.936	
Ramachandran plot statistics (%)		
Preferred	93.61	
Allowed	6.16	
Outlier	0.23	
